# Spinal pain in pre-adolescence and the relation with screen time and physical activity behavior

**DOI:** 10.1186/s12891-021-04263-z

**Published:** 2021-04-26

**Authors:** Anne Cathrine Joergensen, Katrine Strandberg-Larsen, Per Kragh Andersen, Lise Hestbaek, Anne-Marie Nybo Andersen

**Affiliations:** 1 Section of Epidemiology, Department of Public Health, Faculty of Health and Medical Science, University of Copenhagen, Oster Farimagsgade 5, Box 2099, DK-1014 Copenhagen K, Denmark; 2 Section of Biostatistics, Department of Public Health, Faculty of Health and Medical Science, University of Copenhagen, Copenhagen, Denmark; 3 Department of Sport Science and Clinical Biomechanics, University of Southern Denmark, Odense, Denmark; 4 Nordic Institute of Chiropractic and Clinical Biomechanics, Odense, Denmark

**Keywords:** Adolescence health, Back pain, Epidemiology, Lifestyle behavior, Musculoskeletal disorder, Prevention, Public health, Screen time, Physical activity

## Abstract

**Background:**

To investigate how screen time and physical activity behavior were associated with spinal pain in pre-adolescence.

**Methods:**

This study included 45,555 pre-adolescents who participated in the 11-year follow-up of the Danish National Birth Cohort. The 11-year follow-up included self-reported information on computer and TV behavior, aspects of physical activity, as well as frequency and intensity of spinal pain (neck-, mid back- and low back pain). Data were linked with parental socioeconomic data from Statistics Denmark registers. Associations were estimated using multinomial logistic regression models. To account for sample selection, we applied inverse probability weighting.

**Results:**

Duration of screen time was stepwise associated with the degree of spinal pain. Compared with those spending < 2 h/day in front of a screen, screen time of ≥6 h/day was associated with a substantially increased relative risk ratio (RRR) of severe pain for both girls (RRR: 2.49, 95% CI: 2.13–2.92) and boys (RRR: 1.95, 95% CI: 1.65–2.32). Being physical inactive was likewise associated with higher likelihood of severe spinal pain (RRR: 1.22, 95% CI: 1.10–1.34) relative to those being moderately active. We observed that being physically active was seemingly associated with lower risk of spinal pain among boys with high frequency of screen time.

**Conclusion:**

Findings indicate that both duration of screen time and physical inactivity are correlated with spinal pain in pre-adolescents with the strongest associations for screen time. Reducing screen time or increasing physical activity might help preventing spinal pain in pre-adolescents, particularly among high frequent screen users. Future prospective studies investigating the causal relationship are necessary.

**Supplementary Information:**

The online version contains supplementary material available at 10.1186/s12891-021-04263-z.

## Introduction

Spinal pain (i.e., neck, mid back and/or low back pain) constitutes a health burden already from the age of 10 [1]. Epidemiological studies aiming to clarify and understand the etiology of spinal pain in children are scarce, but factors such as symptoms of depression [2, 3], stress and poor general well-being [4], as well as living in socioeconomically disadvantaged families [1] and being exposed to pain in early postnatal life [5] have been suggested as potential risk factors.

Screen-based activities have become ubiquitous components in most families and in educational settings. This fuels young people with an extensive amount of daily screen time, displacing time from a more active lifestyle. Screen-based activities poses different postural demands on the spine; however, common for the activities is nevertheless the static and rigid bodily postures relative to the screen, resembling ergonomic stressors [6–8]. Accordingly, recent and smaller studies have linked different types of sedentary activities such as computers, digital games, TV’s, tablet and smartphones with musculoskeletal outcomes, including spinal pain, in adolescents [9–13].

The World Health Organization (WHO) recommends children to engage in at least 60 min of moderate-to-vigorous activity daily to improve health, including musculoskeletal health, and to prevent non-communicable diseases [14]. Evidence regarding the impact of physical activity behaviors on the development of spinal pain is contradicting [15–19]. Some studies suggest no association [16, 17]. Two prospective studies pointed toward a u-shaped distribution as moderate intensity of physical activity had a potential protective effect, whereas inactivity and vigorous activity (i.e., highly intensive sports activities) increased the risk of back pain [15, 20]. On the contrary, high level of physical activity was also suggested to protect against low and mid back pain in early adolescence [18].

It is plausible that the emergence of screen-based activities has introduced a new risk factor for pre-adolescence spinal pain. Naturally it follows that time spent on screen-based activities displaces time from being physically active since there is limited hours of daily leisure time, and thereby eliminating the potential protective effect of a physically active lifestyle in regard to spinal pain [21]. Increased understanding of the interplay between screen-based activities and physical activity behavior is crucial for policy makers in their design of polices and interventions to approach poor lifestyle behaviors; thus, preventing negative health outcomes. Therefore, we aimed to investigate how time spent on screen-based activities and physical activity behavior were associated with spinal pain in pre-adolescents and further to evaluate the potential heterogenous effect of screen time on spinal pain across levels of physical activity using data collected in the large-scale Danish National Birth Cohort (DNBC).

## Methods

### Study participants

**Fig. 1:**
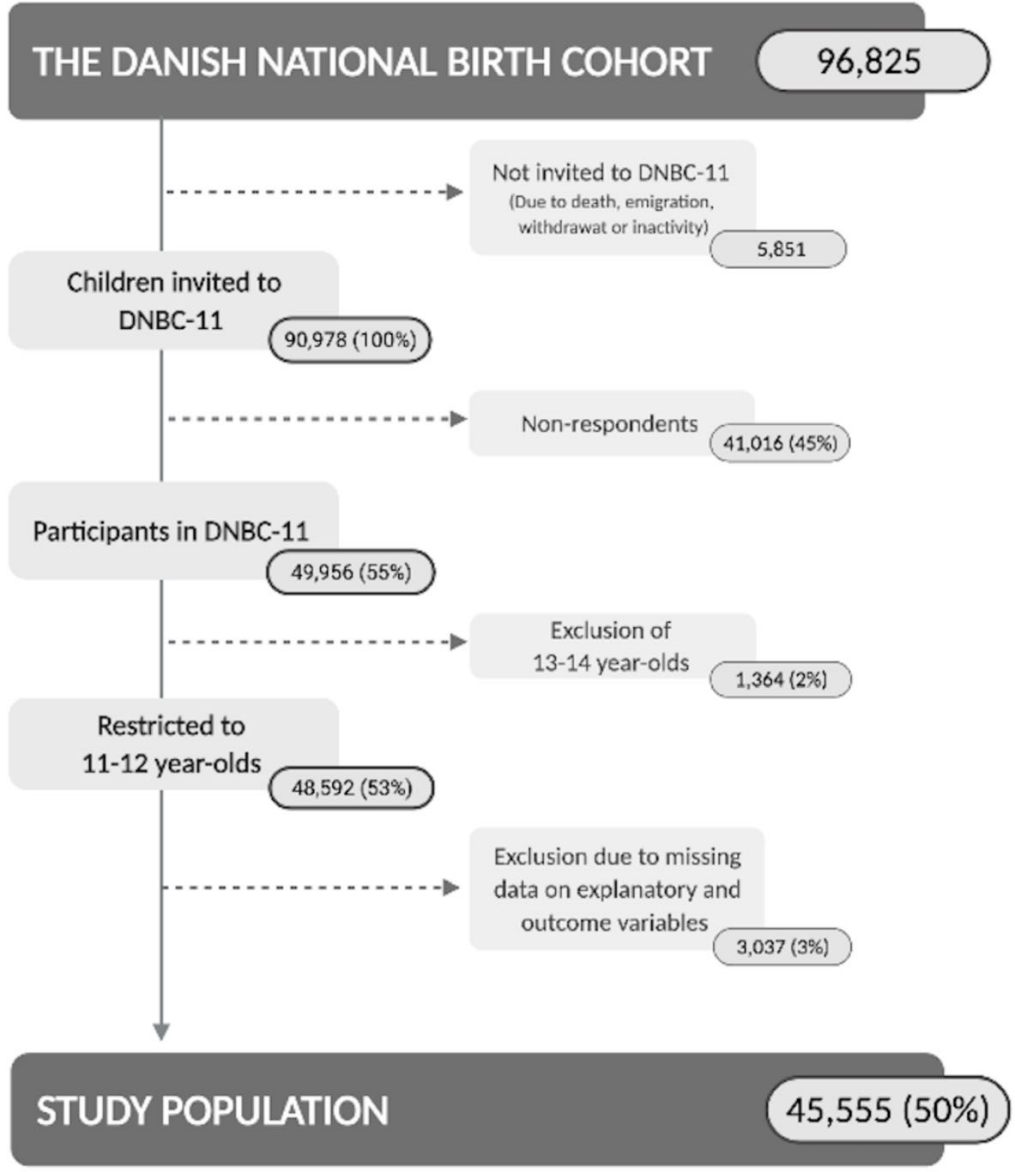
Flow chart of the eligible study population from all liveborn children in The Danish National Birth Cohort

This study was conducted as a cross-sectional study using data from children participating in the 11-year follow-up (DNBC-11) of DNBC. DNBC is a population-based cohort of children born in Denmark from 1996 through 2003. Children and their mothers were followed with several follow-ups from pregnancy and through childhood to young adulthood [22]. DNBC-11 were carried out in the period from July 2010 to September 2014, where children received a web-based self-administered questionnaire to respond from home around their 11th birthday. The questionnaire was pilot tested in focus-group interviews for content validity of each question and functionality. Further details of DNBC and DNBC-11 are described elsewhere (www.dnbc.com) [22]. The study population in the present study consisted of 45,555 11–12-year-olds that participated in DNBC-11 and provided full information on explanatory and outcomes measures (Fig. 1).

All registries applied were available and processed in Statistics Denmark and were linked to DNBC-data through the unique personal identification number assigned to all persons with a permanent residence in Denmark (CPR-number) [23]. However, no personal identifiable data were accessible.

### Screen time

In DNBC-11 the children were asked to report how much of their leisure time they were spending 1) in front of the computer, 2) on computer gaming (i.e., on computer, PlayStation, XBox, PSP, Nintendo and Wii), and 3) watching TV/films. All questions were reported separately for weekdays and weekend days. For weekdays the response categories were given on a seven-point scale ranging from less than ½ hour to more than 5 h, and for weekend days on a 10-point scale ranging from less than ½ hour to more than 8 h (Exact phrasing can be found on www.dnbc.dk). Since item 1 and 2 were not mutually exclusive, we created two different exposure variables. For the main analyses, we defined daily screen time by summing hours spent in front of the computer and reported hours spent on TV watching. We calculated the total daily average by assigning weights to the estimates of weekdays and weekend days (weekdays 5/7, weekend days 2/7). Based on this continuous variable, we constructed the following categorical variable consisting of four consecutive groups: *< 2 h/day, 2 to < 4 h/day, from 4 to < 6 h/day,* ≥ *6 h/day.* In addition to this variable, we constructed a secondary definition of screen time based on time spent with computer gaming etc. and TV watching. This variable was generated similar to the main variable.

### Physical activity behavior

We created two variables for physical activity behavior based on self-reported information regarding general activity level in school breaks and in leisure time, engagement in organized sport activities in leisure time and biking to and from school. Our definition of physical activity behavior was based on WHO’s definition of physical activity among children and young people. WHO’s definition includes activities such as games, playing, sports, transportation, recreation, physical education and planned exercise in the context of family, school and community activities, and also recommends that physical activities should accumulate minimum 60 min daily [14].

Our main variable was categorized into four consecutive groups: *Inactive; lightly active; moderately active; vigorously active*. Due to power limitations in interaction analyses, we simplified the physical activity variable into a binary index categorized as *physically active* vs. *physically inactive*. The inactive group in the binary variable remained identical to the inactive group in the categorical variable and represented children that in general were defining themselves as being inactive during the day. Exhaustive explanation of the variables and the definition of the different categories can be found in Additional file 1.

### Self-reported spinal pain

DNBC-11 included a sub-division of The Young Spine Questionnaire (YSQ) [24]. The YSQ is designed to measure neck, mid and low back pain in 9–11 year-olds with questions on pain frequency (often/once in a while/once or twice/never) and intensity (from 1:“No pain” to 6:” Very much pain” based on the Faces Pain Scale-Revised (FPS-r)) [24, 25]. For each spinal region we combined pain frequency and intensity and trichotomized into *no pain, moderate pain or severe pain.* For all spinal regions, s*evere pain* was defined as pain of 4 or more on FPS-r and occurring at least ‘once in a while’, and *no pain* was defined as pain of 0, 1 or 2 on FPS-r occurring ‘once in a while’ or ‘once or twice’. Moderate pain covered the remaining combinations. Subsequently, *spinal pain* was constructed as a composite measure including all spinal regions categorized according to severity. The applied definition is directly adopted from our previous work on spinal pain and exhaustively explained and illustrated in the study by Joergensen et al. 2019 [1] in which prevalence of spinal pain for the same population can be explored.

### Covariates

A priori, we selected potential confounders, identified using the methods of causal diagrams [26] and supported by experience from our previous work [1].

Information on child’s sex, age and family type was derived from DNBC-11. Parity was obtained from The Danish Medical Birth Registry [27]. Information on parental education was obtained from The Danish Population’s Education Register [23]. Educational level was operationalized as the highest completed education of the parents attained the year of the child’s 11th birthday and was categorized into three groups according to the International Standard Classification of Education (ISCED) 2011: low (ISCED 0–2), medium (ISCED 3–4) and high (ISCED 5–8) [28]. Equivalized household income the year of the child’s 11th birthday was based on disposable household income extracted from The Income Statistics Register [29]. We divided disposable household income by an equivalence factor corresponding to the modified OECD scale [30]. This enabled comparison of family income across family size and composition. We further categorized equivalized household income into quartiles by year relative to all mothers giving birth in the given year.

### Statistics

We applied multinomial logistic regression models to calculate crude and adjusted relative risk ratios (RRR) and corresponding 95% confidence intervals (CI) for the association between screen time and physical activity behavior, respectively, and spinal pain. In all analyses, children with no spinal pain were considered the reference outcome. The dependency between siblings in the sample (*n* = 6076) was taken into account by applying a robust standard error estimator [31]. All statistical analyses were performed in STATA V. 16.1.

Due to the assumption that screen and physical activity behavior may differ between boys and girls and since girls report more spinal pain, we a priori decided to explore sex-differences evaluating first-order interactions between child’s sex and screen time, and between child’s sex and physical activity using Wald-tests. Since the tests indicated interaction for screen time (*P* = 0.059), but not for physical activity (*P* = 0.877), we included the interaction between child’s sex and screen time together with the main effects of physical activity in model 2, and in model 3 we further adjusted for the covariates listed above.

We evaluated the possible heterogenous effect of screen time on spinal pain across levels of physical activity by Wald-testing the three-way interaction between sex, screen time and physical activity behavior. Further, we made a ‘loss to follow-up analysis’ to evaluate whether selection forces in regard to participation in DNBC-11 may have biased our results [32]. Additionally, we used inverse probability weighting (IPW) to account for the study population being a selected sample of the source population [33]. For the latter, we used a reference population consisting of all children born in Denmark between 1996 and 2003 and alive at their 11th birthday (*n* = 526,194). The probability of participation in the study was estimated for each individual using a given set of variables predicting selection into the cohort and loss to follow-up. These factors included parental education at birth, equivalised household income the year before birth, parity, urbanization, maternal smoking during pregnancy and maternal age at birth. All these variables were obtained from Statistic Denmark and therefore available for all participants as well as non-participants. We hereto estimated a weight for each child (i.e., the inverse of the probability of selection) such that each participant was representing not only him/herself but also children with similar characteristics that did not participate in the study. Further, instead of excluding all the individuals for which some of the prediction variables were missing, we estimated the weights based on the best possible set of existing prediction variables.

Lastly, we examined the robustness of the results by conducting a number of sensitivity analyses. Firstly, we performed analyses using neck, mid and low back pain as separate outcomes. Secondly, we used our secondary definition of screen time, and thirdly, we restricted the study population to only include 11-year-olds to accommodate potential age trends. Lastly, since concerns have been raised regarding the importance of mental health [4, 34], we decided to do a sub-analysis in which we adjusted for general wellbeing and stress in children.

## Results

Almost half of the study participants spent 2 to < 4 h/day on screen time, whereas 22% spent < 2 h/day, and almost 9% spent ≥6 h/day (Table 1). This distribution varied slightly between boys and girls i.e., boys spent more time than girls. Study participants spending gradually more time on screens daily differed from their peers spending < 2 h/day with characteristics pointing toward lower socio-economic status, as well as they had siblings and did not live with both of their parents (Table 1). Similar patterns were seen for decreasing level of physical activity (Additional file 1). Comparing the two screen time definitions showed that girls spent more time in front of the computer (i.e., homework etc.) than with computer gaming (Additional file 2).

**Table 1:** Characteristics of the 45,555 pre-adolescents included in the study population according to the main exposure status (screen-based activity) (11-year follow-up, The Danish National Birth Cohort, 1996–2003)

		Average time spent on screen-based activity (h/day)^a^			
Characteristics	N	< 2	2 to < 4	4 to < 6	≥ 6
Total^b^	45,555 (100)	10,104 (22.2)	22,057 (48.4)	9452 (20.8)	3942 (8.7)
Physical activity behavior					
Inactive	5201 (11.4)	753 (7.5)	2251 (10.2)	1366 (14.5)	831 (21.6)
Lightly active	20,436 (44.9)	4186 (41.5)	9730 (44.1)	4544 (48.1)	1973 (49.3)
Moderately active	17,853 (39.2)	4519 (44.7)	9065 (41.1)	3218 (34.1)	1051 (26.7)
Vigorously active	2065 (4.5)	643 (6.4)	1011 (4.6)	324 (3.4)	87 (2.2)
Sex					
Boys	21,711 (47.7)	3686 (36.5)	10,177 (46.1)	5355 (56.7)	2493 (63.2)
Girls	23,844 (52.3)	6418 (63.5)	11,880 (53.9)	4097 (43.4)	1449 (36.8)
Age					
11 years	38,326 (84.1)	8943 (88.5)	18,784 (85.2)	7599 (80.4)	3000 (76.1)
12 years	7229 (15.9)	1161 (11.5)	3273 (14.8)	1853 (19.6)	942 (23.9)
Parental educational level^c^					
High	30,629 (67.2)	7389 (73.1)	14,994 (68.10)	5987 (63.3)	2259 (57.3)
Medium	14,100 (31.0)	2579 (25.5)	6699 (30.4)	3276 (34.7)	1546 (39.2)
Low	826 (1.8)	136 (1.4)	364 (1.7)	189 (2.0)	137 (3.5)
Equivalised household income^c^					
4th quartile (highest)	16,333 (35.9)	4082 (40.4)	8095 (36.7)	3055 (32.3)	1101 (27.9)
3rd quartile	14,011 (30.8)	2996 (29.7)	6848 (31.1)	3010 (31.9)	1157 (29.4)
2nd quartile	10,225 (22.4)	2006 (19.9)	4836 (22.0)	2259 (23.9)	1124 (28.5)
1st quartile (lowest)	4986 (11.0)	1020 (10.1)	2278 (10.3)	1128 (12.0)	560 (14.2)
Family type					
Living with both parents	35,638 (78.2)	8110 (80.3)	17,449 (79.1)	7193 (76.1)	2886 (73.2)
Not living with (both) parents^d^	9917 (21.8)	1994 (19.7)	4608 (20.9)	2259 (23.9)	1056 (26.8)
Parity^e^					
Nulliparous	21,976 (48.2)	5498 (54.4)	10,755 (48.6)	4152 (43.9)	1571 (39.9)
Parous	23,579 (51.8)	4606 (45.6)	11,302 (51.4)	5300 (56.1)	2371 (60.2)

^a^ Variables were analyzed with the chi-squared test of heterogeneity. Chi-squared tests were statistically significant for all variables

^b^ For total distribution of screen-based activity row percentage is shown; for all the covariates column percentages are shown

^c^ Measured the year of the child’s 11th birthday

^d^ Parents not living together due to divorce, separation, they never lived together or only one parent alive

^e^ Maternal parity status in index pregnancy

### Association between screen time, physical activity behavior and spinal pain

The risk ratio of having moderate or severe spinal pain at age 11–12 relative to no pain increased stepwise with increasing screen time (Table 2). The associations were only slightly attenuated subsequent to adjustment for potential confounding (Table 2, model 3). Compared with those spending < 2 h/day on screen time, screen time of ≥6 h/day was associated with a substantial increased risk ratio of reporting severe pain for both girls (RRR: 2.49, 95% CI: 2.13–2.92) and boys (RRR: 1.95, 95% CI: 1.65–2.32). The associations appeared stronger for severe than for moderate pain among those spending ≥6 h/day on screen time. Analysis of physical activity indicated a u-shape for severe spinal pain. Comparing with the moderately active pre-adolescents (RRR: 1.00), lightly (RRR: 1.09, 95% CI: 1.02–1.16) and vigorously active (RRR: 1.09, 95% CI: 1.02–1.16) had similar increased risk ratio, while inactive pre-adolescents had the highest likelihood of spinal pain (RRR: 1.22, 95% CI: 1.10–1.34) (Table 2).

**Table 2:** Relative risk ratio (RRR) of spinal pain according to screen time and physical activity among the 45,555 pre-adolescents included in the study population (The Danish National Birth Cohort, born 1996–2003)

		Model 1^ab^		Model 2^bc^		Model 3^bd^	
	No. of cases Moderate/Severe	Moderate pain RRR (95% CI)	Severe pain RRR (95% CI)	Moderate pain RRR (95% CI)	Severe pain RRR (95% CI)	Moderate pain RRR (95% CI)	Severe pain RRR (95% CI)
Screen time, girls							
< 2 h/day	1734/728	Ref.	Ref.	Ref.	Ref.	Ref.	Ref.
2 to < 4 h/day	3651/1584	1.25 (1.17–1.34)	1.30 (1.18–1.43)	1.24 (1.16–1.33)	1.28 (1.16–1.41)	1.23 (1.15–1.32)	1.24 (1.13–1.37)
4 to < 6 h/day	1372/631	1.49 (1.37–1.63)	1.64 (1.45–1.84)	1.47 (1.34–1.61)	1.59 (1.41–1.80)	1.43 (1.31–1.57)	1.49 (1.32–1.68)
≥ 6 h/day	493/332	1.80 (1.58–2.05)	2.89 (2.48–3.38)	1.76 (1.54–2.01)	2.77 (2.37–3.24)	1.69 (1.48–1.93)	2.49 (2.13–2.92)
Screen time, boys							
< 2 h/day	973/283	Ref.	Ref.	Ref.	Ref.	Ref.	Ref.
2 to < 4 h/day	2856/922	1.11 (1.02–1.22)	1.24 (1.07–1.43)	1.11 (1.01–1.21)	1.23 (1.06–1.41)	1.10 (1.01–1.20)	1.21 (1.05–1.39)
4 to < 6 h/day	1652/553	1.31 (1.19–1.44)	1.51 (1.29–1.76)	1.29 (1.17–1.41)	1.47 (1.26–1.71)	1.26 (1.15–1.39)	1.41 (1.20–1.64)
≥ 6 h/day	761/355	1.38 (1.23–1.55)	2.21 (1.87–2.62)	1.34 (1.19–1.50)	2.11 (1.78–2.51)	1.30 (1.16–1.46)	1.95 (1.65–2.32)
Physical activity behavior							
Inactive	1627/769	1.25 (1.16–1.33)	1.56 (1.42–1.71)	1.14 (1.06–1.22)	1.28 (1.16–1.41)	1.12 (1.04–1.20)	1.22 (1.10–1.34)
Lightly active	6225/2474	1.14 (1.09–1.19)	1.20 (1.12–1.28)	1.11 (1.06–1.16)	1.12 (1.05–1.20)	1.09 (1.04–1.14)	1.09 (1.02–1.16)
Moderately active	5066/1914	Ref.	Ref.	Ref.	Ref.	Ref.	Ref.
Vigorously active	574/231	0.98 (0.88–1.09)	1.04 (0.90–1.21)	1.00 (0.90–1.11)	1.09 (0.94–1.26)	1.00 (0.90–1.11)	1.08 (0.93–1.26)

^a^ Crude model (Screen time was analyzed as the interaction between screen time and child’s sex)

^b^ Reference categories: For explanatory variables; less than 2 h of daily screen time and being physically active, and for outcome variables; not having reported moderate or severe spinal pain in DNBC-11 (No pain)

^c^ Simultaneously modelled for the interaction between screen time and child’s sex, and physical activity behavior, but without further adjustments

^d^ Adjusted for child’s age, parity, family type, parental education, household income and simultaneously modelled for the interaction between screen time and child’s sex, and physical activity behavior

Modeling screen time and physical activity behavior simultaneously (Table 2, model 2), the relative changes in estimates compared with those from the crude analyses were negligible for screen time, but more pronounced for physical inactivity, suggesting that the effect of physical activity on spinal pain may be partially explained by screen time.

**Fig. 2:**
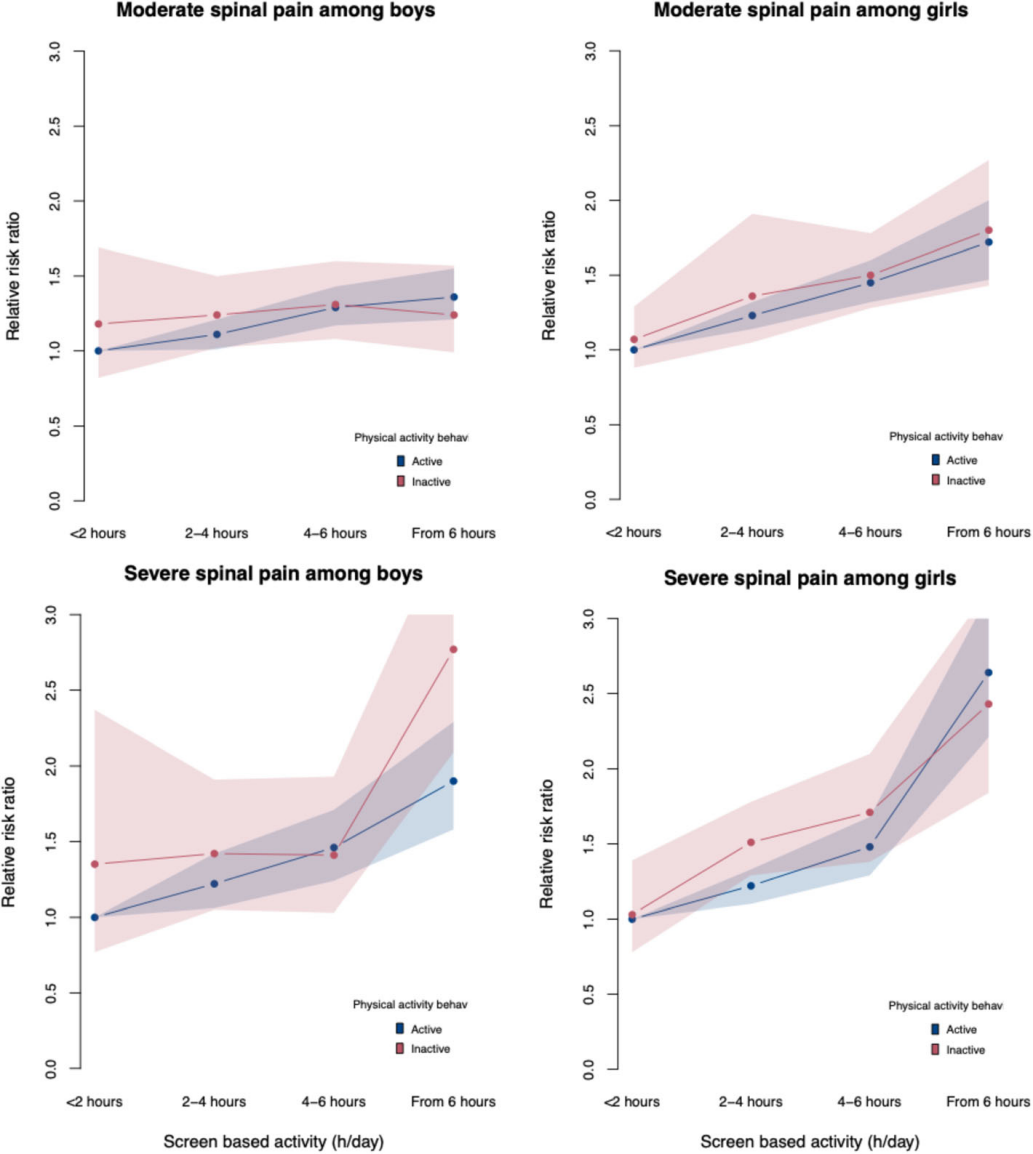
Plots of interaction between child’s sex, screen time and physical activity behavior on spinal pain in pre-adolescents (*N* = 45,555). Test of interaction: *P* = 0.08. Adjusted for child’s age, parity, family type, parental education and household income

Indication of interaction between child’s sex, screen time and physical activity was observed (*P* = 0.08), showing that being physically active was seemingly associated with lower risk of severe spinal pain among boys with high frequency of screen time (Fig. 2).

No remarkable differences were revealed when investigating the spinal regions separately (Additional file 4, 5 and 6), nor when applying the secondary screen time definition (Additional file 7), or when restricting the analytical sample to include 11-year-olds only (Additional file 8). However, when adjusting for general well-being and stress in children, the associations were diminished for screen time and almost removed for physical activity (Additional file 9).

### Selection of study participants

Pre-adolescents lost to follow-up constituted 50% and their characteristics pointed toward lower socio-economic status, not being first-born and living outside urban areas (Additional file 10). However, the weighted analyses in which we applied IPW to account for selection both into to the cohort and from attrition showed no essential changes to the estimates (Additional file 11).

## Discussion

In this study of more than 45,000 pre-adolescents from DNBC, we demonstrated that increasing screen time were stepwise associated with increased likelihood of both moderate and severe spinal pain in pre-adolescents. Further, physically inactive pre-adolescents were more likely to report severe spinal pain than the more active pre-adolescents, and the associations were indicative for a u-shape. Lastly, we demonstrated that being physically active was seemingly associated with lower risk of spinal pain among boys with high frequency of screen time.

Our results complement the previous literature showing that the risk of spinal pain in children and adolescents increases monotonously with increasing hours spent in front of a screen [9–11]. We further showed that the association with screen time was independent of physical activity. This is in line with two studies indicating that the association between sedentary activities and physical complaints in young people is not attributable to physical activity behavior [11, 12], and a meta-analysis suggesting sedentary and physical activity to be separate constructs that independently of each other impact on health outcomes [35]. We did, however, observe that some of the crude effect of physical inactivity was attributable to screen time, but not vice versa.

For severe spinal pain, we observed tendencies of a u-shaped association with physical activity. A smaller-scale Danish prospective study, with accelerometer measurements of physical activity, showed that shifting from inactive to moderate intensity of activities tended to protect against spinal pain while vigorous physical activity increased the risk of developing spinal pain [19]. Increased risk of developing spinal pain was likewise observed in the 10% most active adolescents in a school-based prospective study [36]. In other studies, the u-shape has not been replicated [16, 17]. Besides differences in methods of measurement which may challenge direct comparisons of study results, the lack of a clear negative impact of vigorously activity on spinal pain may be explained by limitations in our data that obstructing us from distinguishing pre-adolescents performing highly intensive sport disciplines, presumably being those at highest risk [15, 19, 36]. However, from a public health perspective, these results suggest that the intensity of physical activity is important for the relationship between physical activity behavior and spinal pain in pre-adolescence.

As screen-based activities and physical activity behavior both may cause or take part in the development of spinal pain [6, 12, 18], we expected to find a heterogenous effect of screen time on spinal pain across the levels of physical activity; however, this was only the case for severe spinal pain among boys. The lack of a clear interaction between the two lifestyle behaviors is likely due to differential impact of sedentary and physical behavior on musculoskeletal health i.e., different impact on different spinal regions.

We have previously with DNBC-11 showed that pre-adolescents with low general wellbeing or high stress levels had higher odds of spinal pain in a cross-sectional design [4]. Therefore, we included the two measures as additional confounders in a sensitivity analysis, which diminished the association with screen time and almost removed the association for physical activity. However, since the temporality is highly uncertain, and wellbeing, stress and spinal pain in principle can be expressions of the same thing i.e., feeling bad, we believe that such adjustment would implicate overadjustment.

The mechanisms whereby sedentary and physical activity contribute to the development of spinal pain remains somewhat unknown. For example, spending a high amount of time sedentary displaces time from a more active lifestyle which may result in overweight as well as affecting sleep quality [37–39]. Both problematic sleep and BMI > 25 Kg/m^2^ have previously been suggested to be associated with the development of musculoskeletal pain [40–44]. However, since physical inactivity and screen time are modifiable behaviors, there seems to be an urgent need for effective population-based strategies.

### Strengths and limitations

The large sample size resulted in stable estimates and sufficient numbers in each of the exposure categories. A further strength is the application of YSQ to measure spinal pain. This instrument has previously been validated among Danish children of 9–11 years of age, and proven to have applicability in cross-sectional studies [24]. Lastly, DNBC is nested within the Danish population; thus, we were able to make individual linkage to health and social data on pre-adolescents and their parents from Danish nationwide registries enabling adjustment for important potential confounders and further to analyze non-participation according to the source population and to attrition.

Limitations include the cross-sectional design and self-reported screen time and physical activity behavior. The cross-sectional design obstructs examination of temporality. One could argue that pre-adolescents with spinal pain refrain from being physically active and thus, spending more time in front of a screen and vice versa. Hence, the study was restricted to investigate the contemporary correlations in the data, exclusively, and no conclusive statements concerning causal directions of the associations have been made.

Misclassification of self-reported sedentary and physical behavior is a general problem in studies on this issue [11, 45, 46]. In this study, the items were only pilot tested for content validity and functionality, but not further tested for reliability and concurrent validity. Moreover, mutually exclusiveness was not given, as the activity across media types might overlap in time which we were not able to completely account for with the collected data [11]. Further, parallel to the DNBC-11 data collection, the iPad was launched. Additionally, in 2011 50% of all Danish 10–12 year-olds had a smartphone, and this percentage has increased rapidly since 2011 [47]. Due to tablets being strong, integrated components in families and educational settings today, the emergence of tablets during and subsequent to the period of the data collection (2010–2014) might have resulted in a shift in screen-environment among children. In addition, the general use of electronic devices has likewise increased rapidly over the last decade. Therefore, the prevalence estimates of screen time might be higher today than by the time DNBC-11 was carried out. Additionally, the applied variable of physical activity behavior was based on a combination of volume, intensity, context and children’s perception of being active defined in line with WHO’s recommendations for physical activity among children and young people [14]. However, the current WHO recommendation of a minimum of 60 daily minutes of moderate to vigorous activities could only be approximated. Despite this, we believe that the applied definition in this work constituted a more compliant measure for this age group than would a proxy based exclusively on attendance in sport activities.

Finally, selection bias cannot be neglected as DNBC-participants are a selected sample of the source population [32]. However, we accounted for sample selection by applying IPW [33, 48], and the weighted results did not reveal any essential changes to the estimates; therefore, we do not consider selection bias as a major problem for the study findings [32, 49].

## Conclusion

Findings indicate that duration of screen time and physical inactivity are correlated with spinal pain in pre-adolescents with the strongest associations for screen time. Reducing screen time or increasing physical activity might help preventing spinal pain in pre-adolescents, particularly among high frequent screen users. Future prospective studies investigating the causal relationship between screen time, physical activity and development of spinal pain in children and adolescents are necessary as further understanding of the interplay is of great public health relevance.

## Supplementary Information

**Additional file 1.**

**Additional file 2.**

**Additional file 3.**

**Additional file 4.**

**Additional file 5.**

**Additional file 6.**

**Additional file 7.**

**Additional file 8.**

**Additional file 9.**

**Additional file 10.**

**Additional file 11.**

## Data Availability

All data were accessed and analyzed via a safe remote access connection to Statistics Denmark. Availability of these data is restricted to research institutions with approved license such as the Danish public universities. The applied data in this study are therefore not publicly available. Access to data can be given upon reasonable request and with permission from Statistics Denmark.

## Abbreviations

DNBC: The Danish National Birth Cohort
DNBC-11: The 11-year follow-up of The Danish National Birth Cohort
FPS-r: Faces Pain Scale-Revised
IPW: Inverse probability weighting
ISCED: International Standard Classification of Education
RRR: Relative risk ratio
Spinal pain: Neck pain, mid back pain and/or low back pain
YSQ: The Young Spine Questionnaire
95% CI: 95% confidence intervals
