# Supplementary material for: Spinal pain in pre-adolescence and the relation with screen time and physical activity behavior
Source: BMC Musculoskelet Disord. 2021 Apr 26;22:393. doi: 10.1186/s12891-021-04263-z (PMC8077847; doi:10.1186/s12891-021-04263-z)

Additional file 1: Explanation of physical activity behavior
To define physical activity behavior, we used several relevant questions from the 11-year-follow-up of The Danish National Birth Cohort
(link to questionnaire https://www.dnbc.dk/-/media/arkiv/projekt-sites/dnbc/kodeboeger/11-year-follow-up-data-documentation/code-book-for-child---in-english.pdf?la=en)

To define general activity behavior, we used the following two questions:

1) *How do you usually use your body during the school breaks? Think about the last months when choosing your answer.*
2) *How do you usually use your body during your leisure time? Think about the last months when choosing your answer.*

The response categories were as follows:

1. *I am very active: I run around or play ball most of the time.*
2. *I am pretty active: I run around and play ball some of the time.*
3. *I walk around during most breaks.*
4. *I sit and talk, read or play games during most breaks.*

To define weekly sport activity, we used the question: "*Do you do sports in your leisure time? Yes/No*" in combination with questions on type of sport and number of times a week they did the different sport types. This was summed, and categorized into; *1-2 times weekly, 3-5 times weekly, minimum 6 times weekly.*

Lastly, we created a variable for biking more or less than 30 minutes daily to and from school.

The variables were combined as follows from the flow chart and categorized info four consecutive groups of physical activity behavior (see figure below).

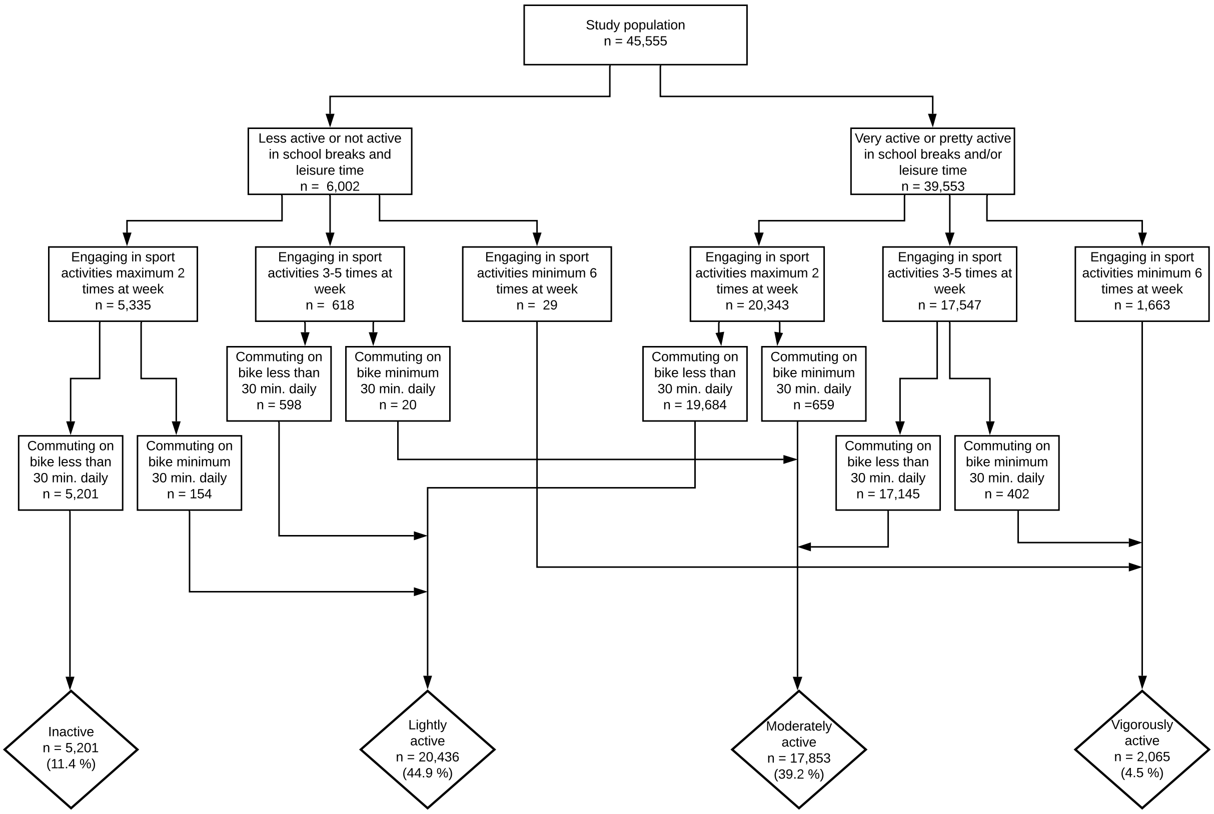

Supplement: Supplementary file 1 — Additional file 1. [file 12891_2021_4263_MOESM1_ESM.pdf]
