# Supplementary material for: Spinal pain in pre-adolescence and the relation with screen time and physical activity behavior
Source: BMC Musculoskelet Disord. 2021 Apr 26;22:393. doi: 10.1186/s12891-021-04263-z (PMC8077847; doi:10.1186/s12891-021-04263-z)
Supplement: Supplementary file 2 — Additional file 2. [file 12891_2021_4263_MOESM2_ESM.pdf]

Additional file 2

Characteristics of the 45,555 pre-adolescents included in the study population according to physical activity behavior (11-year follow-up, The Danish National Birth Cohort, born 1996-2003)

Characteristics	Physical activity behavior[a]			
	Inactive (%)	Lightly active (%)	Moderately active (%)	Vigorously active (%)
Total[b]	11.4	44.9	39.2	4.5
SBA (h/day)				
< 2	14.5	20.5	25.3	31.1
2 to < 4	43.3	47.6	50.8	49.0
4 to < 6	26.3	22.2	18.0	15.7
≥ 6	16.0	9.7	5.9	4.2
Sex				
Boys	33.3	47.2	51.7	54.0
Girls	66.7	52.8	48.3	46.0
Age				
11 years	82.0	85.7	83.8	77.7
12 years	18.0	14.3	16.2	22.3
Parental educational level[c]				
High	63.9	65.1	70.1	71.7
Medium	33.3	32.5	28.9	27.4
Low	2.9	2.3	1.0	0.9
Equivalised household income[c]				
4th quartile (highest)	28.9	32.3	41.0	44.0
3rd quartile	29.5	30.4	31.5	30.8
2nd quartile	27.3	24.5	19.2	17.4
1st quartile (lowest)	14.3	12.7	8.3	7.9
Family type				
Living with both parents	71.7	75.2	82.6	87.0
Not living with (both) parents[d]	28.3	24.8	17.4	13.0
Parity				
Nulliparous	50.5	48.2	48.2	44.0
Parous	49.5	51.8	51.9	56.0

a Variables were analyzed with the chi-squared test of heterogeneity. Chi-squared tests were statistically significant for all variables

b For total distribution of physical activity behavior, row percentage is shown; for all the covariates column percentages are shown.

c Measured at age 11

d Parents not living together due to divorce, separation, they never lived together or only one parent alive.
