# Supplementary material for: Spinal pain in pre-adolescence and the relation with screen time and physical activity behavior
Source: BMC Musculoskelet Disord. 2021 Apr 26;22:393. doi: 10.1186/s12891-021-04263-z (PMC8077847; doi:10.1186/s12891-021-04263-z)

Additional file 3

Comparison of the distributions of the two SBA definitions according to child's sex. Main definition is based on time in front of the computer and TV viewing, whereas the alternative measure is based on computer gaming and TV viewing.

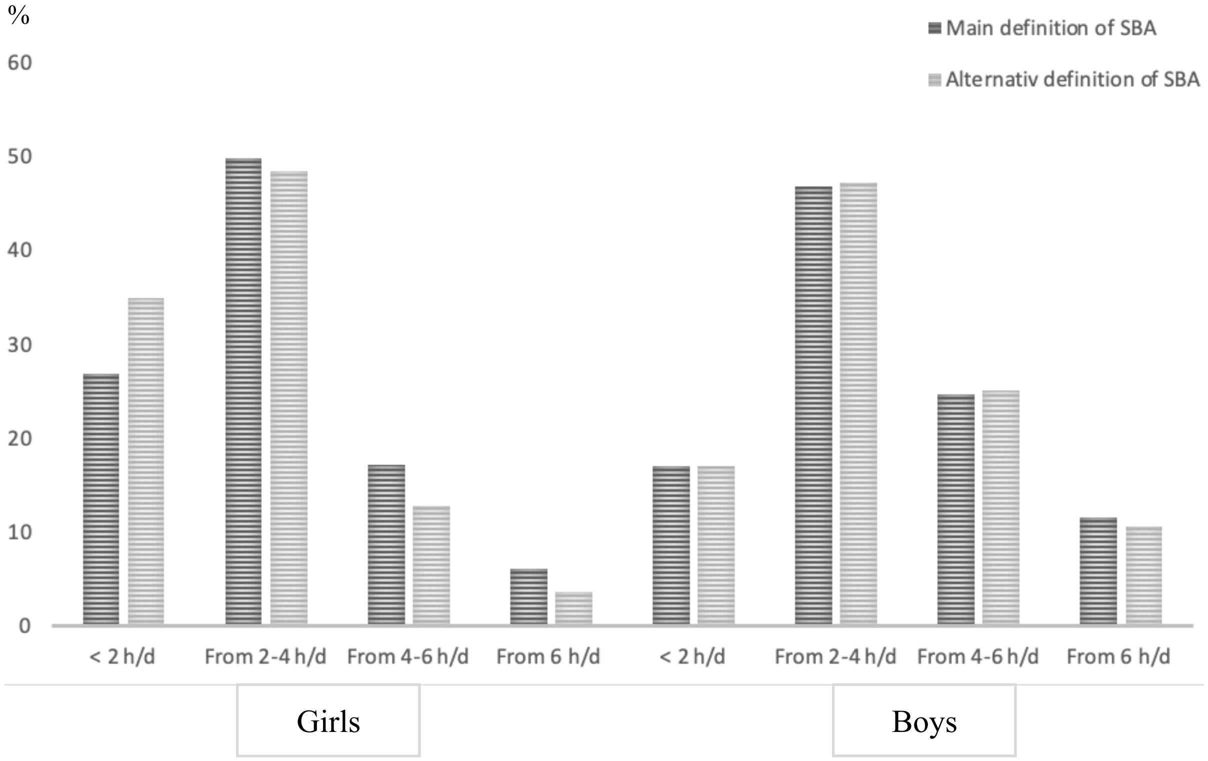

Supplement: Supplementary file 3 — Additional file 3. [file 12891_2021_4263_MOESM3_ESM.pdf]
