# Supplementary material for: Spinal pain in pre-adolescence and the relation with screen time and physical activity behavior
Source: BMC Musculoskelet Disord. 2021 Apr 26;22:393. doi: 10.1186/s12891-021-04263-z (PMC8077847; doi:10.1186/s12891-021-04263-z)
Supplement: Supplementary file 5 — Additional file 5. [file 12891_2021_4263_MOESM5_ESM.pdf]

Additional file 5

Relative risk ratio (RRR) of mid back pain according to screen-based activity and physical activity, respectively, among the 45,555 pre-adolescents included in the study population (The Danish National Birth Cohort, born 1996-2003)

		Model 1[ab]		Model 2[bc]		Model 3[bd]	
	**No. of cases**	**Moderate pain**	**Severe pain**	**Moderate pain**	**Severe pain**	**Moderate pain**	**Severe pain**
	Moderate/Severe	RRR (95% CI)	RRR (95% CI)	RRR (95% CI)	RRR (95% CI)	RRR (95% CI)	RRR (95% CI)
SBA, Girls (h/day)							
< 2	797/249	Ref.	Ref.	Ref.	Ref.	Ref.	Ref.
2 to < 4	1,619/556	1.12 (1.03-1.23)	1.24 (1.06-1.44)	1.11 (1.02-1.22)	1.22 (1.05-1.42)	1.09 (1.00-1.20)	1.18 (1.01-1.37)
4 to < 6	671/235	1.42 (1.27-1.59)	1.59 (1.32-1.91)	1.38 (1.24-1.55)	1.54 (1.28-1.85)	1.33 (1.19-1.49)	1.42 (1.18-1.71)
≥ 6	278/140	1.82 (1.56-2.12)	2.93 (2.36-3.64)	1.75 (1.51-2.04)	2.78 (2.23-3.47)	1.65 (1.42-1.93)	2.45 (1.96-3.06)
SBA, Boys (h/day)							
< 2	397/86	Ref.	Ref.	Ref.	Ref.	Ref.	Ref.
2 to < 4	1,246/288	1.16 (1.03-1.31)	1.24 (0.97-1.59)	1.15 (1.02-1.30)	1.23 (0.96-1.57)	1.14 (1.01-1.29)	1.21 (0.95-1.55)
4 to < 6	777/189	1.43 (1.26-1.63)	1.60 (1.24-2.08)	1.39 (1.22-1.58)	1.56 (1.20-2.03)	1.36 (1.19-1.55)	1.49 (1.15-1.93)
≥ 6	382/150	1.57 (1.35-1.83)	2.85 (2.17-3.74)	1.50 (1.29-1.75)	2.71 (2.06-3.56)	1.44 (1.23-1.68)	2.47 (1.88-3.25)
Physical activity behavior							
Inactive	796/298	1.33 (1.22-1.45)	1.65 (1.43-1.89)	1.21 (1.11-1.33)	1.32 (1.14-1.53)	1.18 (1.08-1.29)	1.25 (1.08-1.44)
Lightly active	2,927/859	1.21 (1-14-1.28)	1.17 (1.05-1.30)	1.17 (1.10-1.24)	1.09 (0.98-1.21)	1.15 (1.08-1.22)	1.04 (0.94-1.16)
Moderately active	2,185/662	Ref.	Ref.	Ref.	Ref.	Ref.	Ref.
Vigorously active	259/74	1.03 (0.90-1.18)	0.97 (0.76-1.14)	1.05 (0.91-1.20)	1.01 (0.79-1.30)	1.05 (0.91-1.20)	1.01 (0.79-1.29)

a Crude model (SBA was analyzed as the interaction between SBA and child's sex)

b Reference categories: For explanatory variables; less than 2 hours used on SBA daily and being moderately physically active, and for outcome variables; not having reported moderate or severe mid back pain in DNBC-11 (No pain)

c Simultaneously modeled for the interaction between SBA and child's sex, and physical activity behavior, but without further adjustments.

d Adjusted for child's age, parity, family type, parental education, household income and simultaneously modeled for the interaction between SBA and child's sex, and physical activity behavior

Test of interaction between SBA and physical activity: P = 0.029
