# Supplementary material for: Spinal pain in pre-adolescence and the relation with screen time and physical activity behavior
Source: BMC Musculoskelet Disord. 2021 Apr 26;22:393. doi: 10.1186/s12891-021-04263-z (PMC8077847; doi:10.1186/s12891-021-04263-z)
Supplement: Supplementary file 9 — Additional file 9. [file 12891_2021_4263_MOESM9_ESM.pdf]

Additional file 9

Relative risk ratio (RRR) of spinal according to screen-based activity and physical activity, respectively, among the 45,555 pre-adolescents included in the study population, with further adjustment for general wellbeing and Stress in Children scale. (The Danish National Birth Cohort, born 1996-2003)

		**Model 1[ab]**		**Model 2[ac]**	
	**No. of cases** Moderate/Severe	**Moderate pain** RRR (95% CI)	**Severe pain** RRR (95% CI)	**Moderate pain** RRR (95% CI)	**Severe pain** RRR (95% CI)
SBA, Girls (h/day)					
< 2	1,434/466	Ref.	Ref.	Ref.	Ref.
2 to < 4	3,053/928	1.20 (1.11-1.28)	1.19 (1.08-1.31)	1.20 (1.12-1.29)	1.19 (1.08-1.32)
4 to < 6	1,135/392	1.35 (1.23-1.48)	1.34 (1.19-1.52)	1.34 (1.22-1.47)	1.30 (1.15-1.48)
≥ 6	445/200	1.55 (1.36-1.77)	2.11 (1.80-2.48)	1.49 (1.30-1.70)	1.94 (1.64-2.28)
SBA, Boys (h/day)					
< 2	781/177	Ref.	Ref.	Ref.	Ref.
2 to < 4	2,247/616	1.07 (0.98-1.18)	1.15 (1.00-1.33)	1.07 (0.98-1.17)	1.14 (0.99-1.32)
4 to < 6	1,345/376	1.20 (1.18-1.40)	1.30 (1.11-2.08)	1.18 (1.07-1.30)	1.25 (1.07-1.47)
≥ 6	640/226	1.21 (1.07-1.36)	1.73 (1.45-2.06)	1.15 (1.02-1.30)	1.56 (1.31-1.87)
Physical activity behavior					
Inactive	1,356/475	1.02 (0.95-1.10)	1.03 (0.93-1.14)	0.98 (0.91-1.06)	0.96 (0.87-1.06)
Lightly active	5,145/1,598	1.06 (1.01-1.11)	1.04 (0.97-1.11)	1.05 (1.01-1.10)	1.02 (0.95-1.09)
Moderately active	4,107/1,162	Ref.	Ref.	Ref.	Ref.
Vigorously active	472/146	1.02 (0.92-1.14)	1.12 (0.97-1.31)	1.02 (0.92-1.13)	1.12 (0.97-1.31)

a Reference categories: For explanatory variables; less than 2 hours used on SBA daily and being moderately physically active, and for outcome variables; not having reported moderate or severe neck pain in DNBC-11 (No pain)

b Adjusted for general wellbeing, child's age, parity, family type, parental education, household income and simultaneously modeled for the interaction between SBA and child's sex, and physical activity behavior

c Adjusted for Strees in Children scale, child's age, parity, family type, parental education, household income and simultaneously modeled for the interaction between SBA and child's sex, and physical activity behavior
