# Supplementary material for: Spinal pain in pre-adolescence and the relation with screen time and physical activity behavior
Source: BMC Musculoskelet Disord. 2021 Apr 26;22:393. doi: 10.1186/s12891-021-04263-z (PMC8077847; doi:10.1186/s12891-021-04263-z)
Supplement: Supplementary file 10 — Additional file 10. [file 12891_2021_4263_MOESM10_ESM.pdf]

Additional file 10

Characteristics of the 90,978 participants invited to participate in DNBC-11 and included in the baseline cohort according to follow-up status at age 11

Characteristics	**Study population** 45,555 (50.1)	**Lost to follow-up**[a] 45,423 (49.9)
Sex		
Boys	21,711 (47.7)	20,942 (46.1)
Girls	23,844 (52.3)	24,458 (53.9)
Missing	0	25 (0.1)
Parental educational level[b]		
High	30,629 (67.2)	23,761 (52.3)
Medium	14,100 (31.0)	19,551 (43.1)
Low	826 (1.8)	2,105 (4.6)
Missing	0	6 (0.1)
Equivalised household income[b]		
4th quartile (highest)	16,333 (35.9)	12,130 (27.0)
3rd quartile	14,011 (30.8)	12,201 (27.1)
2nd quartile	10,225 (22.4)	11,865 (26.4)
1st quartile (lowest)	4,986 (11.0)	8,789 (19.5)
Missing	0	438 (1.0)
Parity		
Nulliparous	21,976 (48.2)	20,721 (45.7)
Parous	23,579 (51.8)	24,642 (54.3)
Missing	0	60 (0.1)
Urbanization[c]		
Copenhagen	6,433 (14.1)	5,387 (11.9)
Sub-urban Copenhagen	4,888 (10.7)	4,866 (10.7)
Provincial cities	6,715 (14.7)	5,816 (12.8)
Provincial towns	13,203 (29.0)	13,599 (30.0)
Rural areas	14,270 (31.3)	15,675 (34.6)
Missing	46 (0.1)	81 (0.2)

a We used chi-squared tests of heterogeneity to compare study participants with individuals lost to follow-up. Chi-squared tests were statistically significant for all variables

b Measured at the year of the child's 11th birthday

c Urbanization registered at childbirth
