# Supplementary material for: Spinal pain in pre-adolescence and the relation with screen time and physical activity behavior
Source: BMC Musculoskelet Disord. 2021 Apr 26;22:393. doi: 10.1186/s12891-021-04263-z (PMC8077847; doi:10.1186/s12891-021-04263-z)
Supplement: Supplementary file 11 — Additional file 11. [file 12891_2021_4263_MOESM11_ESM.pdf]

Additional file 11

Relative risk ratio (RRR) of spinal pain according to time spent on SBA and physical inactivity among the 45,555 pre-adolescents included in the study population and weighted according to a reference population consisting of all children born in Denmark from 1996-2003 (N = 526,194)

	**Model 1**[abc]		**Model 2**[bcd]		**Model 3**[bce]	
	**Moderate pain** RRR (95% CI)	**Severe pain** RRR (95% CI)	**Moderate pain** RRR (95% CI)	**Severe pain** RRR (95% CI)	**Moderate pain** RRR (95% CI)	**Severe pain** RRR (95% CI)
SBA, Girls (h/day)						
< 2	Ref.	Ref.	Ref.	Ref.	Ref.	Ref.
2 to < 4	1.25 (1.15-1.35)	1.29 (1.15-1.45)	1.24 (1.14-1.34)	1.28 (1.14-1.43)	1.22 (1.13-1.33)	1.23 (1.10-1.38)
4 to < 6	1.46 (1.32-1.62)	1.62 (1.40-1.86)	1.44 (1.30-1.60)	1.57 (1.36-1.81)	1.40 (1.26-1.55)	1.45 (1.26-1.68)
≥ 6	1.80 (1.53-2.10)	2.98 (2.46-3.60)	1.77 (1.51-2.07)	2.86 (2.36-3.46)	1.68 (1.43-1.97)	2.54 (2.10-3.07)
SBA, Boys (h/day)						
< 2	Ref.	Ref.	Ref.	Ref.	Ref.	Ref.
2 to < 4	1.12 (1.02-1.24)	1.27 (1.09-1.49)	1.12 (1.01-1.23)	1.26 (1.08-1.47)	1.11 (1.00-1.23)	1.24 (1.06-1.46)
4 to < 6	1.33 (1.19-1.48)	1.60 (1.34-1.90)	1.31 (1.17-1.46)	1.56 (1.31-1.85)	1.28 (1.14-1.43)	1.49 (1.25-1.78)
≥ 6	1.43 (1.25-1.64)	2.38 (1.96-2.90)	1.39 (1.21-1.60)	2.27 (1.86-2.76)	1.34 (1.17-1.54)	2.09 (1.71-2.55)
Physical activity behavior						
Inactive	1.19 (1.10-1.29)	1.57 (1.41-1.76)	1.08 (1.00-1.18)	1.28 (1.13-1.42)	1.05 (0.97-1.14)	1.18 (1.05-1.33)
Lightly active	1.13 (1.08-1.20)	1.24 (1.15-1.34)	1.10 (1.04-1.16)	1.16 (1.07-1.25)	1.08 (1.02-1.14)	1.11 (1.02-1.19)
Moderately active	Ref.	Ref.	Ref.	Ref.	Ref.	Ref.
Vigorously active	0.94 (0.83-1.06)	1.04 (0.87-1.25)	0.96 (0.85-1.08)	1.09 (0.91-1.31)	0.96 (0.85-1.08)	1.09 (0.91-1.30)

a Crude model (SBA was analyzed as the interaction between SBA and child's sex)

b Reference categories: For explanatory variables; less than 2 hours used on SBA daily and being moderately physically active, and for outcome variables; not having reported moderate or severe spinal pain in DNBC-11 (No pain)

c Inverse probability weighing. Applied variables to predict selection both into the cohort and loss to follow-up encompassed parental education at birth, equivalised household income the year before birth, parity, urbanization, maternal smoking during pregnancy and maternal age at birth

d Simultaneously modeled for the interaction between SBA and child's sex, and physical activity behavior, but without further adjustments

e Adjusted for child's age, parity, family type, parental education, household income and simultaneously modeled for physical activity behavior and the interaction between SBA and child's sex
